# Technique-specific reduction of sciatic nerve stiffness through neurodynamic stretches at submaximal and maximal intensity in asymptomatic individuals

**DOI:** 10.1007/s00421-026-06320-8

**Published:** 2026-06-29

**Authors:** Marion Hitier, Denis César Leite Vieira, Carole Cometti, Joao Luiz Quagliotti Durigan, Nicolas Babault

**Affiliations:** 1 Université Bourgogne Europe, INSERM, CAPS UMR 1093, 21000 Dijon, France; 2 Sport Science Faculty, Centre d’Expertise de la Performance, Université Bourgogne Europe, BP 27877, 21078 Dijon Cedex, France; 3 Chiropractor, Private Practice, 21000 Dijon, France; 4 Laboratory of Muscle and Tendon Plasticity, Graduate Program in Rehabilitation Sciences, Universidade de Brasília, Brasília, DF Brazil; 5 College of Physical Education, University of Brasilia, Brasília, DF Brazil

**Keywords:** Neural tissue, Shear wave elastography, Stiffness, Stretching intensity, Range of motion

## Abstract

**Purpose:**

To compare the immediate effects of two neurodynamic mobilization techniques (flossing vs. tensioning) on the sciatic nerve and hamstring muscle tissues, using different stretching intensities.

**Methods:**

Twenty physically active volunteers performed a randomized, study comprising seven conditions (six stretching + control). Interventions included static stretching (SS), neurodynamic tensioning (TENS), and neurodynamic flossing (FLOS), applied at 100% (pain threshold) or 90% maximal range of motion, using 5 × 60-s sets. Shear wave elastography (SWE) was used to assess sciatic nerve and biceps femoris stiffness before and immediately after the interventions. Measurements included flexibility tests (passive knee extension (PKE), stand and reach (SR), slump), and hamstring strength (MVIC) alongside with electromyography (EMG).

**Results:**

SWE of the sciatic nerve showed a significant condition × time interaction (p < 0.001), with a significant decrease only after flossing at maximal intensity (FLOS100); The biceps femoris (BF) shear wave velocity (SWV) decreased over time (p = 0.004) independently of the condition. Flexibility outcomes significantly improved after all stretching interventions (p < 0.001). Nevertheless, FLOS100 produced significantly greater improvements in sciatic nerve mobility than SS at maximal intensity (p = 0.017). MVIC significantly decreased from PRE to POST (p = 0.009) across all conditions, with no condition × time interaction (p = 0.991). The EMG activity remained unchanged for BF muscle while semitendinosus (ST) EMG showed a significant decrease over time (p = 0.017).

**Conclusions:**

Neurodynamic flossing at maximal intensity reduced sciatic nerve stiffness and mobility in asymptomatic individuals. These neural adaptations suggest flossing should be prioritized for neural tissue, while all stretching techniques similarly affect muscle stiffness.

## Introduction

Neurodynamic mobilization techniques are widely applied in rehabilitation and physiotherapy to enhance the mobility and function of peripheral nerves, particularly in the management of neuropathic pain such as carpal tunnel syndrome, radiculopathies, or sciatica (Ballestero-Pérez et al. 2017; Neto et al. 2019). For example, for the sciatic nerve, it consists of associating proximal and distal joint movements (i.e. around the cervical and ankle joint) while stretching the hamstring muscles. These interventions are designed to act on the mechanics and physiology of neural tissue by reducing nerve mechanosensitivity, therefore increasing motor conduction velocity (Basson et al. 2017; Zaheer and Ahmed 2023), and optimizing nerve sliding through the surrounding interfaces (Coppieters and Butler 2008). Two main neurodynamic approaches exist: nerve tensioning, which involves maintaining the nerve stretched at the end of the joint range of motion with relatively limited excursion, and nerve flossing (also termed gliding or sliding), which consists of alternating proximal and distal joint movements to induce greater neural sliding while avoiding excessive tensile stress (Coppieters et al. 2009; Ellis et al. 2017).

These techniques are commonly employed in the rehabilitation of peripheral neuropathies (Bittencourt et al. 2024). For example, a previous study has demonstrated positive mechanical effects in patients with sciatica, who often present alterations in the mechanical properties of the sciatic nerve (Neto et al. 2019). Three minutes of slump mobilization, i.e. a form of neurodynamic flossing in a seated position (Maitland and Maitland), was efficient to reduce sciatic nerve stiffness in chronic patients (Neto et al. 2019), suggesting acute mechanical plasticity of the neural tissue. Nonetheless, in healthy individuals, these three minutes of slump mobilization did not induce any immediate reduction of the sciatic nerve stiffness (Neto et al. 2019). In contrast, others reported an acute decrease in sciatic nerve stiffness in healthy volunteers after maintaining a six-minute slump position (Andrade et al. 2018). These contradictory findings highlighted the current lack of consensus regarding the acute effects of the different possible neurodynamic techniques on sciatic nerves, particularly in healthy tissues.

Although neurodynamic mobilizations are efficient for clinical patients, these techniques could be of interest in healthy individuals and more particularly in athletes. Notably, recent meta-analyses demonstrated that neurodynamic techniques acutely improve hamstring flexibility in asymptomatic individuals, with a higher efficiency over static stretching in short-term applications (De Arenas-Arroyo et al. 2025). Moreover, authors have shown neurodynamic mobilizations could enhance flexibility without acutely altering muscle force (Zaheer and Ahmed 2023; Hitier et al. 2025). These findings contrast with the widely used static stretching (Babault et al. 2021) which generally produces transient strength decreases with durations longer than 60 s (Warneke et al. 2025). Additionally, unlike conventional muscle stretching, neurodynamic techniques mobilize not only contractile tissues and fascial interfaces but also neural structures, which may induce specific neural adaptations (Andrade et al. 2018; Heredia et al. 2024). However, neurodynamic mobilizations are scarcely studied in sport settings and little is known about the methodology to be applied.

Stretching intensity, for example, can modulate acute neuromuscular responses (Bryant et al. 2023). Some studies have reported greater gains in range of motion (ROM) with high-intensity stretching (Freitas and Mil-Homens 2015; Nakamura et al. 2021). However, the literature remains elusive since reaching pain threshold was not necessary to improve flexibility (Hatano et al. 2022). In contrast, in clinical practice, neurodynamic stretching is generally performed at a low intensity and adapted to each patient to limit pain and avoid inflammatory reactivation or tissue aggravation (Shacklock 1995). Given these findings, one can question the neurodynamic technique and intensity to be applied in healthy individuals.

The present study aimed to determine the immediate effects of two neurodynamic mobilization techniques (flossing versus tensioning) on sciatic nerve and hamstring muscle tissues using different stretching intensities in healthy, physically active individuals. We employed shear wave elastography (SWE) to quantify tissue stiffness changes, complemented by comprehensive functional assessments. Based on mechanotransduction principles and recent neurophysiological evidence, we formulated two primary hypotheses: (i) neurodynamic flossing at maximal intensity produces greater reduction in sciatic nerve stiffness compared to neurodynamic tensioning at matched intensity and static stretching; and (ii) due to the specific nerve mobilization of neurodynamic techniques, the secondary hypothesis was that submaximal intensity (90% range of motion) would achieve equivalent reductions in sciatic nerve stiffness compared to maximal intensity (100%).

## Materials and methods

### Participants

Twenty volunteers (12 females and 8 males) were recruited. Volunteers had the following characteristics (mean ± standard deviation): age 24.2 ± 2.6 years; height 171.4 ± 10.1 cm; body mass 65.5 ± 9.7 kg and physical activity per week 7.0 ± 3.9 h. The physical activities performed by the participants included a wide range of sports, including resistance training (4), discus throw, running (4), swimming (2), climbing, horseback riding (2), French boxing, team sports (3), and racket sports (2). Inclusion criteria were: over 18 years old, physically active and without having had a lower limb injury or back pain in the past three months, or injury to a specific muscle in the past two years (exclusion criteria). Before participating, they were fully informed about the purpose of the study and experimental procedure. They all signed an informed consent form. This study was conducted according to the Declaration of Helsinki. It was approved by the national sport science ethics committee (CERSTAPS, IRB00012476-2022–15-03–166). To determine the minimum required number of participants, the sample size was calculated a priori using G*Power (version 3.1.9.7) with the following criteria: effect size of 0.40 (large), power of 0.8, probability error of 0.05. The values were chosen based on the existing literature on nerve SWV (our main outcome), which reported a large effect after stretching interventions (Andrade et al. 2018). A sample size of 20 people has been indicated.

### Experimental design

**Fig. 1:**
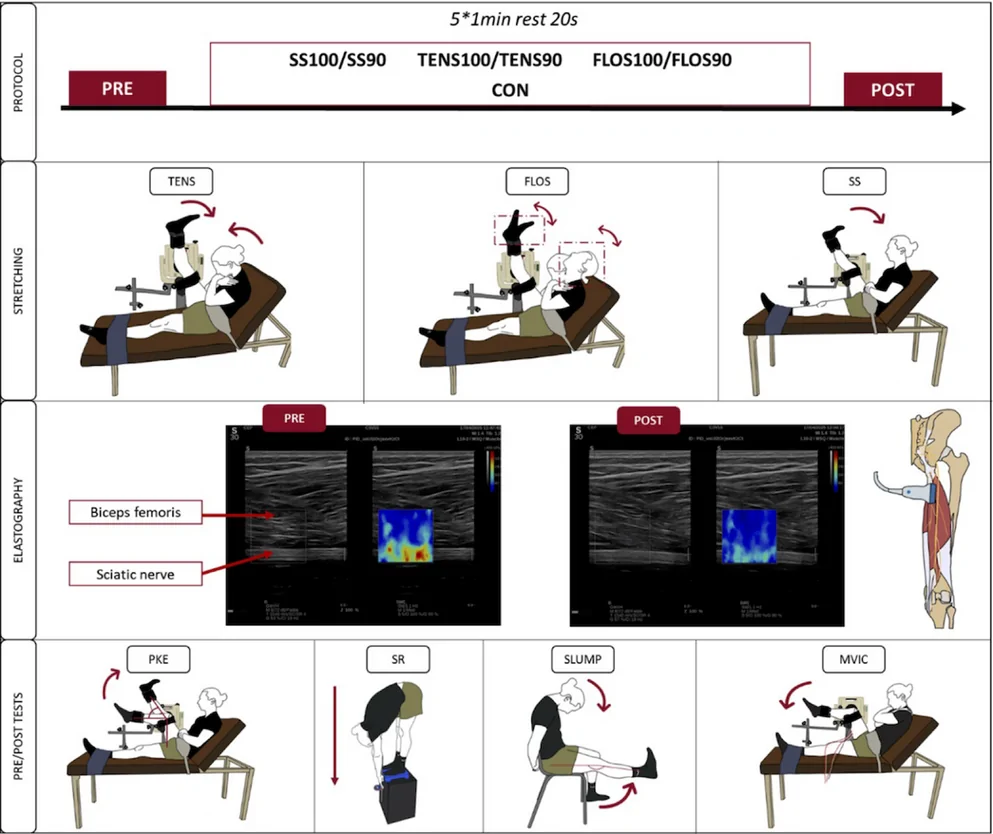
Study experimental design. The timeline, the stretching interventions, the main outcome assessed by shear wave elastography (PRE and POST stretching, with shear wave velocity color mapping and identification of the biceps femoris and sciatic nerve, as well as probe positioning), and all PRE and POST intervention tests are presented. *SS* Static stretching condition; *FLOS* Neurodynamic flossing condition; *TENS* Neurodynamic tensioning condition; *CON* Control condition; *PKE* Passive knee extension test; *SR* Stand and reach test; *MVIC* Maximal voluntary isometric contraction

The study was a randomized, single-blind, crossover and controlled trial. Blinding was for data and statistical analyses. The volunteers attended eight sessions, with an interval of at least 48 h between each session. Sessions took place at the same time of the day. During the study period, the participants were asked to continue with their usual training routine, but to refrain from any intensive exercise for at least two days prior to the experimental test sessions. A familiarization session was first conducted to explain the different experimental procedures, collect anthropometric data (age, height, body mass) and introduce the volunteers to the different stretching techniques, tests and elastography procedure. Then, participants were tested during seven experimental sessions with data collected before and immediately after the different conditions. Tests consisted of SWE to assess shear wave velocity of biceps femoris and sciatic nerve tissues. Afterward, a passive knee extension (PKE) test was performed to determine the maximal ROM. Then, maximal torque was evaluated during two hamstring maximal voluntary isometric contractions (MVIC). Finally, we evaluated the multisegmental flexibility using the Stand and Reach test (SR) and the sciatic nerve mobility using the Slump test. Three experimental sessions involved hamstring stretching at maximal intensity: static stretching (SS100), neurodynamic tensioning stretching (TENS100), and neurodynamic flossing stretching (FLOS100). These stretching modalities were performed at the point of pain corresponding to 100% of the maximal range of motion. The remaining sessions followed the same protocols but with a stretching intensity set at 90% of the maximal range of motion: static stretching (SS90), neurodynamic tensioning stretching (TENS90), and neurodynamic flossing stretching (FLOS90). The last experimental session served as control (CON) (Fig. 1). All experimental sessions were time-matched and presented in a random order using www.randomizer.org. Limb dominance was ignored, and all conditions and tests were conducted on the right knee flexors on an isokinetic dynamometer (Biodex 4 Quickset, Biodex Corporation, Shirley, NY, USA). The temperature of the laboratory was controlled and was statistically similar across all sessions (21.1 ± 0.6 °C).

### Experimental conditions

During the six stretching sessions, participants performed static, neurodynamic tensioning, or neurodynamic flossing (gliding) stretching exercises at 100% (i.e., point of pain) and at 90% (i.e., point of discomfort) of their maximal range of motion. It comprised 5 sets of 60 s, with 20 s of rest between sets (Chaouachi et al. 2017). It was performed with the participants lying supine with the right hip flexed at 110 degrees. Their right hamstrings, ankle, left leg and pelvis were attached to limit other movements.

During static stretching intervention, the right knee was placed at a standardized angle of 90 degrees using an isokinetic dynamometer (Riccetti et al. 2020). The knee was then rotated to reach the point of hamstrings pain (SS100) or 10% below (SS90). The speed was set at 5°.s^−1^ to avoid the myotatic reflex (Kay and Blazevich 2010). The position was maintained for 60 s. Afterward, the dynamometer released the participant’s limb back to a 90° knee angle at a 20°.s^−1^ angular speed. Twenty seconds of rest were provided and then the stretching process was repeated four additional times (Oba et al. 2021). Volunteers were asked to stay relaxed during the stretching and not to offer any resistance.

During the neurodynamic tensioning condition, participants received the same movement from the dynamometer at the point of pain (TENS100) or 10% below (TENS90). However, from the start of the stretching until the release, volunteers were asked to position their right ankle in a maximal dorsiflexion concomitant with their head flexed toward the sternum. This position was similar to the slump test (see below) and aimed to create an elongation of the sciatic nerve bed (Coppieters et al. 2009) (Fig. 1).

During the neurodynamic flossing condition, participants received the same movement from the dynamometer at the point of pain (FLOS100) or 10% below (FLOS90). However, participants were asked to make cranial/caudal movements of their head and right ankle. The instruction was to plantarflex the ankle and flex the head simultaneously, then to dorsiflex the ankle while extending the head. These movements were alternated every 2 s, using an electronic timer (Sharma et al. 2016) (Fig. 1).

During the control condition (CON), volunteers were asked to stay relaxed while keeping their knee at 90° and maintaining their head and ankle in a neutral position. The duration of the protocol (400 s) matched the duration of the stretching protocols.

Although stretching intensity was standardized using the knee angle determined during the PKE test, the additional cervical and ankle movements performed during the neurodynamic conditions likely modified neural strain across modalities. Therefore, “intensity 100%” should be interpreted as a subjective threshold based on the PKE test rather than as a quantitative neural mechanical load between conditions.

### Tests and data analyses

Measurements were conducted before the stretching or control procedures (PRE) and immediately afterwards (POST). Tests duration was about two minutes. More specifically, SWV, MVC, PKE, S-R, and slump tests were performed at 15 s, 30 s, 1 min, 1 min 30 s, and 1 min 50 s after interventions, respectively. Tests started with a resting elastography image of the biceps femoris and the sciatic nerve with the hip maintained at 110° of flexion and the knee at 90°. Images were taken using an Aixplorer ultrasound scanner (Supersonic MACH30, Imagine, v. 6.1, Aix-en-Provence, France), coupled to a linear transducer array (2–10 MHz, SL10-2, Supersonic Imagine, Aix-en-Provence, France). The ultrasound was used in SWE mode (musculoskeletal preset). The maximal scale was set to 400 kPa, with an image depth of 6 mm. This image was used to analyze muscle and sciatic nerve shear wave velocities (SWV) expressed in m.s^−1^ (Bercoff et al. 2004) (Fig. 1). The optimal probe position was determined using B-mode imaging and marked on the skin to ensure measurements’ reproducibility. Consistent position across all sessions was ensured using anatomical markers, found and positioned on the image by the same operator. These markers included the position and orientation on the image of the sciatic nerve and of the muscular fibers of the gluteus maximus and of the biceps femoris. The probe was surrounded by a custom-made device to ensure no pressure was put directly on the probe, therefore maintaining a constant pressure between the probe and the skin surface. As a result, the pressure on the probe was only influenced by the orientation of the device, which was the same between all trials and sessions. The pressure was also controlled using the scaling of the ultrasound image. Two images per testing period were recorded in SWE mode, with the opacity map fixed at 0% to ensure a blinded data collection. A single image was selected for analysis based on its quality and the accuracy of the shear wave measurements (i.e. uncolored pixels) by an assessor blinded to condition and time point. Analysis was performed with the Qbox tool using the elastography software. Seven Qbox regions of interest were placed on the sciatic nerve and seven on the biceps femoris. The mean Qbox value was then calculated separately for the nerve and muscle.

The maximal ROM of the knee was then measured using the PKE method (Gajdosik et al. 1993). The PKE test consisted of passively extending the right knee joint (by the same examiner) from 90° knee flexion to the point of pain (when the participant verbally indicated the onset of pain), after which the knee was immediately returned to the neutral position (90° of flexion). Participants were instructed to maintain their ankle in dorsiflexion and to remain as relaxed as possible. The maximal ROM obtained was used as the reference value for the 100% stretching conditions (SS100, TENS100, and FLOS100). For the 90% conditions (SS90, TENS90, and FLOS90), 10% was subtracted from the maximal ROM to determine the stretching position. To ensure relaxation during this test, the EMG activity was continuously monitored and recorded from the biceps femoris (BF) and semitendinosus (ST) as previously described (Vieira et al.^2021^). No muscle contraction was observed during any of the interventions.

After thirty seconds of rest, volunteers performed two isometric MVIC of the knee flexors at a 90° knee angle. Participants were instructed to contract maximally for 5 s, with a rest interval of 20 s between the two attempts. The trial with the highest torque was selected for data analysis. Due to poor signal quality, data from one participant were not included in the analysis. The maximum knee flexion torque and corresponding RMS values for BF and ST muscles were recorded. EMG activity was recorded using two pairs of silver chloride electrodes (10 mm diameter) with an inter-electrode distance of 2 cm. The skin under the electrodes was previously shaved and dry-cleaned with alcohol to obtain low impedance (< 5 kΩ). Electrodes for the BF were applied at 50% on the line between the ischial tuberosity and the lateral epicondyle of the tibia according to SENIAM recommendations. For ST, electrodes were placed at 50% on the line between the ischial tuberosity and the medial epicondyle of the tibia. The reference electrode was placed on the ipsilateral patella. The EMG signals were amplified with a bandwidth frequency ranging from 10 Hz to 5 kHz (gain = 500). The EMG signals (as well as torque traces) were recorded with a Biopac MP150 system (Biopac System, Santa Barbara, CA) at a sampling frequency of 2 kHz and stored for analysis with AcqKnowledge software (AcqKnowledge 4.2 for MP systems, Biopac System, Santa Barbara, CA). EMG activity during stretching protocol and MVC was quantified using RMS values over 500 ms sliding windows around peak torque value.

Global flexibility was assessed by the stand-and-reach test (Holt et al. 1999). Briefly, participants stood upright with their feet parallel and were then instructed to bend forward as far as possible (Jankowicz-Szymańska et al. 2022). The upper limbs were fully extended (elbow, wrist, and fingers), and the maximum distance reached was measured using a flexometer (TK500, Takei scientific instruments, Niigata, Japan). This procedure was performed once at each time point.

Flexibility and sciatic nerve mobility were assessed using the slump test (Maitland 1985). The participant was seated on a chair with the neck flexed forward and the ankle positioned in dorsiflexion. Simultaneously, the participant extended the right knee. The examiner used an inclinometer (Inclinometer Application, Syleos Apps, version 1.82) to measure the maximal knee extension, placing it on the tibial tuberosity. This measurement corresponded to the angular difference relative to the horizontal position (180°). This procedure was performed once at each time point.

### Statistical analyses

Data were expressed as mean ± standard deviation (SD) or as mean differences with their 95% confidence intervals (95% CI). The normality and sphericity of the data were verified using the Shapiro–Wilk and Mauchly’s tests, respectively. Normality assumptions were also specifically verified for percentage change data prior to the additional analyses using the same methododology. A two-way repeated-measures analysis of variance (ANOVA; Condition × Time) was then conducted. The condition factor corresponded to the seven experimental conditions, and time represented the two time points (PRE and POST). When the assumption of sphericity was violated, the Greenhouse–Geisser and Huynh–Feldt corrections were applied. In case of significant time effect, one-way ANOVA was conducted on percent changes to determine differences between conditions. When significant main effects or interactions were observed, Bonferroni-adjusted post-hoc tests were performed. Partial eta squared (η^2^ₚ) was calculated from the ANOVA results, with thresholds of 0.01, 0.06 and above 0.14 interpreted as small, medium and large effects respectively. The level of statistical significance was set at p < 0.05 for all analyses. All Statistical analyses were performed using JASP software (version 0.95.4, JASP Team 2025, University of Amsterdam, available for free at https://jasp-stats.org/download/).

## Results

Nineteen out of twenty participants completed all experimental sessions; one participant’s EMG data were excluded due to poor signal quality (excessive noise and signal instability). This exclusion consisted only of the EMG data, and all other measurements from this participant were retained for statistical analyses. All 20 participants’ shear wave elastography, flexibility, and torque data were included in the analysis.

### Elastography assessments

**Fig. 2:**
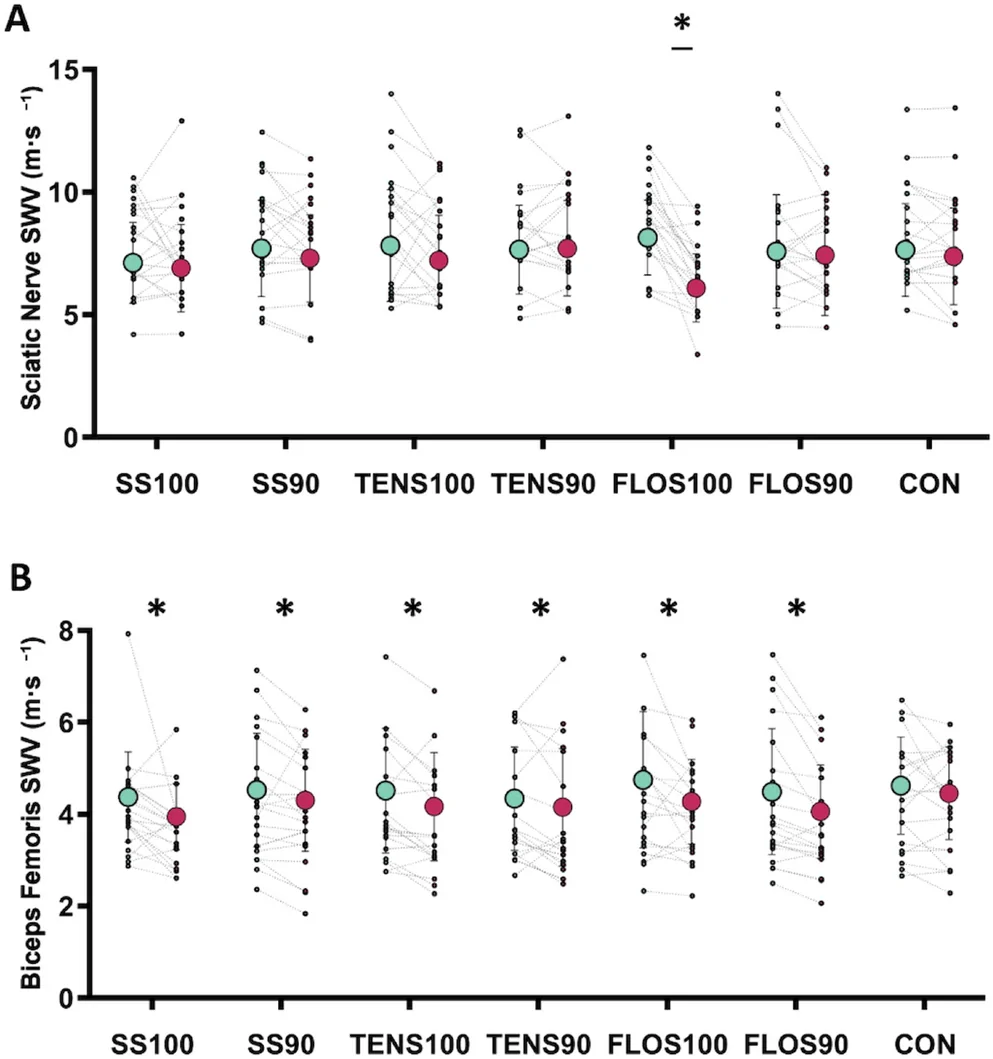
Shear wave velocity (SWV) on the sciatic nerve (**A**), and biceps femoris muscle (**B**). Individual (grey dots) and mean values (green = PRE, and red = POST) of muscle stiffness assessed using shear-wave elastography (SWE) across the experimental conditions are shown. *Significant difference compared to pre (p < 0.001)

SWE on the sciatic nerve did not reveal any difference condition main effect (p = 0.221) (Table 1). A significant time effect (p < 0.001) and a condition × time interactions (p < 0.001) were observed. Post-hoc analyses showed a significant decrease between PRE and POST only for FLOS100 condition (p < 0.001). The percent decrease for FLOS100 was significantly greater compared to the other conditions (p < 0.001) (Fig. 2).

**Table 1:** Results of two-way repeated-measures ANOVA

Variable	Effect	F	p	η^2^_p_
SWE Nerve sciatic	Condition	1.400	0.221	0.069
	Time	18.262	< 0.001**	0.443
	Condition × Time	4.784	< 0.001**	0.201
SWE Biceps femoris	Condition	1.114	0.358	0.055
	Time	10.538	0.004*	0.526
	Condition × Time	0.757	0.605	0.038
Passive knee extension	Condition	0.575	0.749	0.029
	Time	121.163	< 0.001**	0.864
	Condition × Time	7.830	< 0.001**	0.292
Stand-and-reach	Condition	2.459	0.028*	0.115
	Time	37.610	< 0.001**	0.664
	Condition × Time	5.998	< 0.001**	0.240
Slump test	Condition	2.478	0.027*	0.115
	Time	22.013	< 0.001**	0.537
	Condition x Time	0.979	0.443	0.049
Maximal torque	Condition	2.244	0.044*	0.106
	Time	8.507	0.009*	0.309
	Condition × Time	0.135	0.991	0.007
Biceps femoris RMS	Condition	0.763	0.600	0.039
	Time	0.846	0.369	0.043
	Condition × Time	0.525	0.788	0.027
Semitendinosus RMS	Condition	0.533	0.782	0.027
	Time	6.779	0.017*	0.263
	Condition × Time	1.234	0.294	0.061

*SWE* Shear wave elastography; *RMS* Root mean square *p < 0.05; **p < 0.001

### Flexibility assessments

**Fig. 3:**
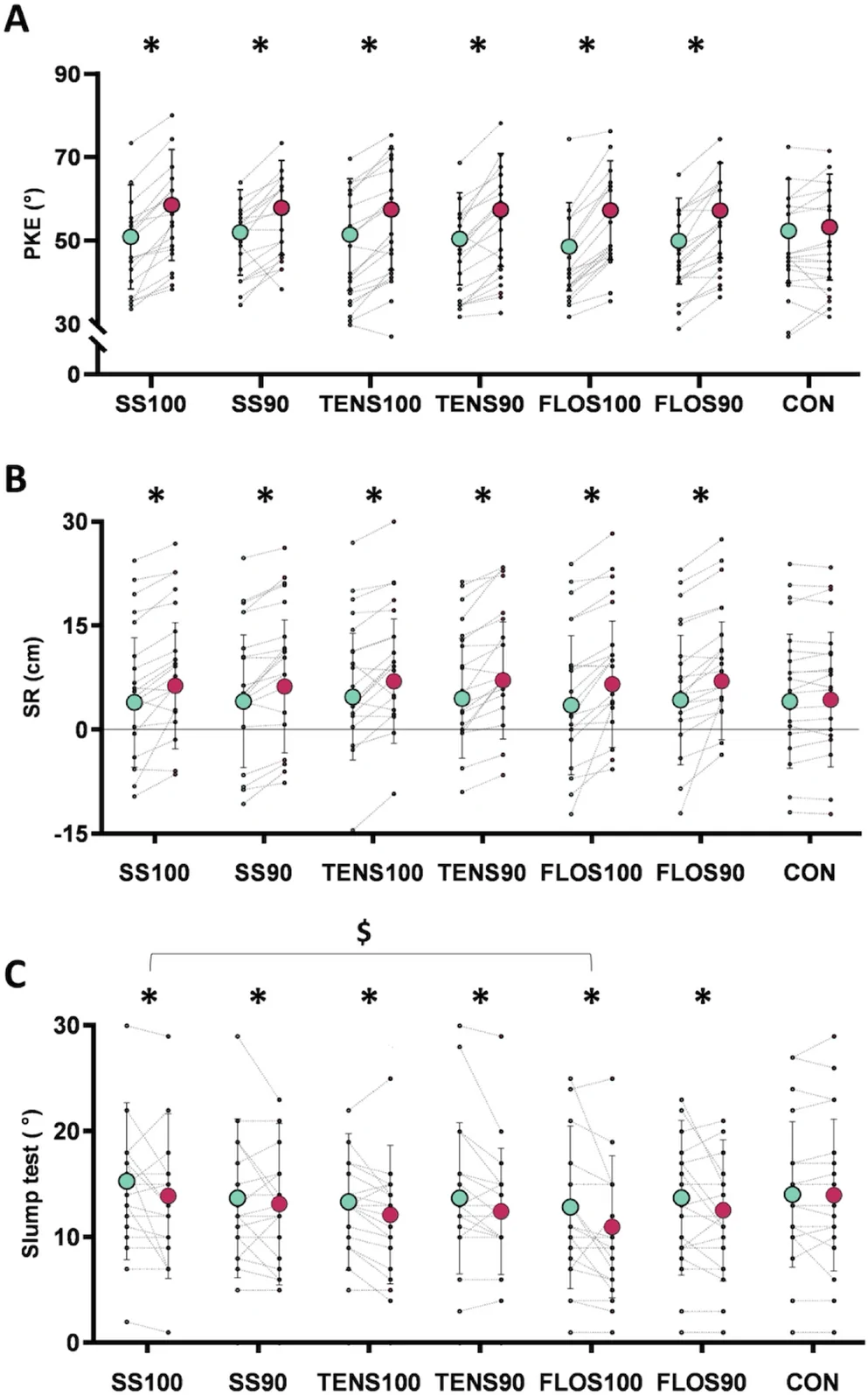
Passive knee extension (**A**), Stand and reach (**B**) Slump test (**C**) measured PRE and POST interventions. Grey dots represent an individual measurement, while colored markers indicate mean values over time (green = PRE, and red = POST). *Significant difference compared to PRE (p < 0.001). $: Significant difference between Floss100 vs SS100 (p < 0.005)

For PKE, no main condition effect was observed (p = 0.749), but there were significant main effect of time (p < 0.001) and significant condition × time interaction (p < 0.001) (Table 1). Post-hoc comparisons indicated significant increases from PRE to POST for all conditions except CON (Fig. 3), with no difference observed between experimental conditions. The PKE alteration was significantly greater in all experimental conditions as compared to CON (p < 0.001).

The SR test demonstrated a significant main effect of condition (p = 0.028), a significant effect of time (p < 0.001) and a significant condition × time interaction (p < 0.001) (Table 1). Post-hoc analyses confirmed significant improvements from PRE to POST for all conditions except CON, while comparisons between experimental conditions were not significant (Fig. 3).

The slump test showed significant differences between conditions (p = 0.027) and time (p < 0.001), without a significant condition × time interaction (p = 0.443) (Table 1). Post-hoc analyses indicated that FLOS100 produced a significantly greater improvement than SS100 (p = 0.017) (Fig. 3), with no other significant difference between conditions.

Due to the important number of tests and sessions, a statistical analysis of the baseline measurements for the SR and slump test of each session was performed. It revealed no significant flexibility × time interaction, which excluded any familiarization effect across sessions.

### Maximal hamstring force production

**Fig. 4:**
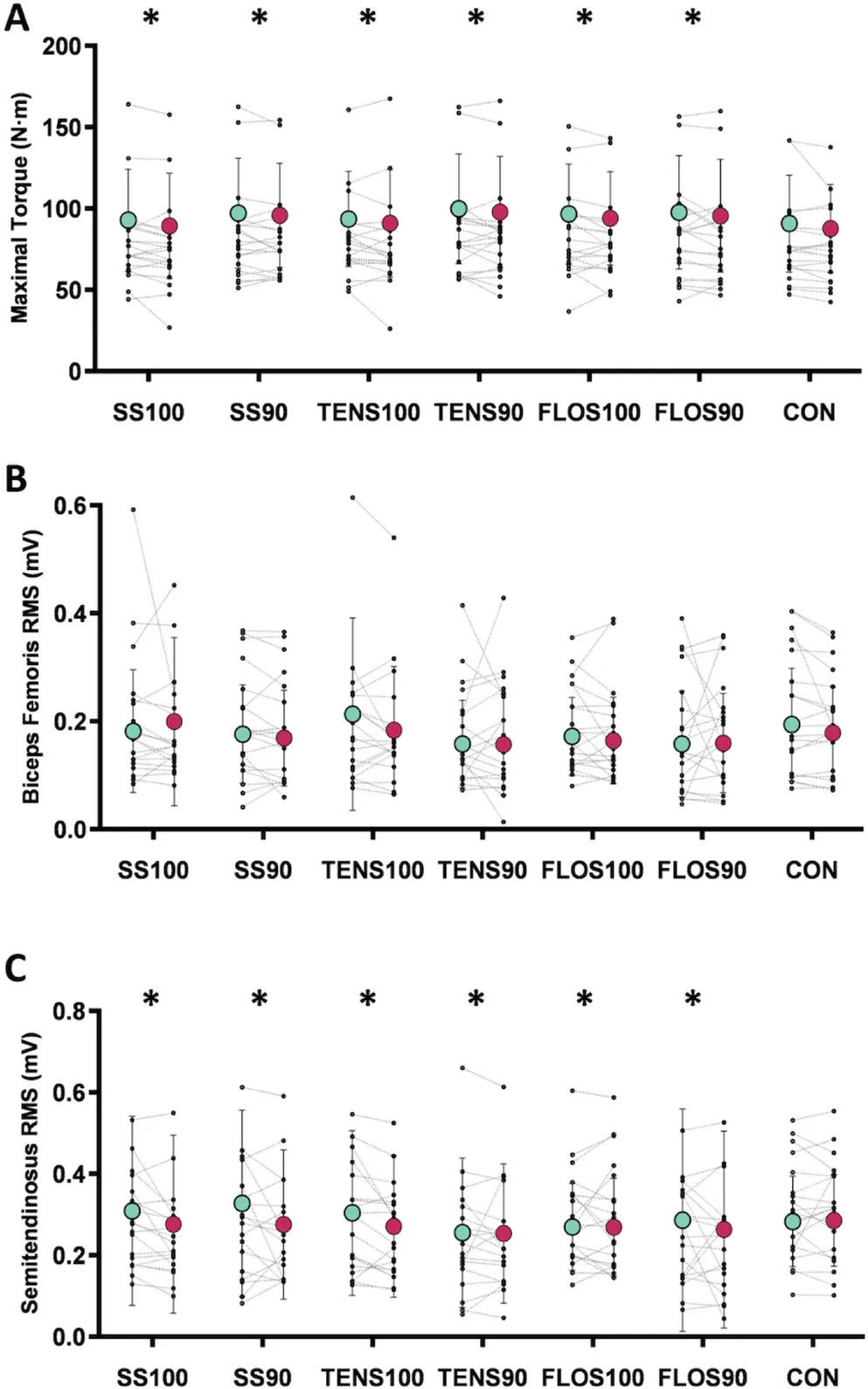
Maximal Torque (**A**), root mean square (RMS), for biceps femoris (**B**) and semitendinosus (**C**) muscles during maximal voluntary isometric contractions. Grey dots represent individual measurements, while colored markers indicate group mean values over time (green = PRE, and red = POST). *Significant difference with respect to POST (p < 0.05)

A significant main effect of time was observed for knee flexor torque (p = 0.009) (Table 1), indicating a significant decrease in force from PRE to POST tests, independently of the experimental condition (Fig. 4). A significant main effect of condition was detected (p = 0.044). without any specific differences observed between conditions accordingly to post-hoc tests (all adjusted p-values > 0.05). No condition × time interaction was found (p = 0.991).

For BF EMG activity, no significant effect was observed for condition (p = 0.600), time (p = 0.369) or condition × time interaction (p = 0.788) (Table 1). For ST, a significant main effect of time was identified (p = 0.017) (Fig. 4). No effect of condition (p = 0.782) or condition × time interaction (p = 0.294) were detected (Table 1). Following post-hoc analyses, the time effect was significant, indicating a decrease in ST EMG activity from PRE to POST tests. No significant change was observed for CON.

## Discussion

This study determined the immediate effects of two neurodynamic mobilization techniques (flossing vs. tensioning) performed at maximal and submaximal intensities on sciatic nerve stiffness, hamstring muscle stiffness, and flexibility in healthy, physically active individuals. The primary finding that maximal-intensity neurodynamic flossing (FLOS100) selectively reduced sciatic nerve stiffness while tensioning and submaximal intensity did not, provides novel mechanistic insight into neurodynamic technique specificity and optimal dosing for asymptomatic neural tissue adaptations.

Stretching is primarily used to improve flexibility by targeting the muscle–tendon complex. Static, dynamic, or proprioceptive neuromuscular facilitation (PNF) stretching modalities are commonly implemented to enhance muscle and joint mobility (Opplert and Babault 2019). In addition, certain techniques, specifically targeting the nervous system, aim to mobilize neural tissues themselves. These techniques include sliders (neural gliding) and tensioners (neural tensioning). Sliders promote nerve excursion through combined joint movements, whereas tensioners apply a global mechanical strain to the nerve (Coppieters et al. 2015). Neurodynamic techniques are primarily used in clinical populations, with numerous beneficial effects reported, particularly for pain reduction. For instance, decreases in pain and functional disability have been observed in individuals with chronic spinal disorders following repeated neurodynamic interventions (Basson et al. 2017). Similarly, reductions in median nerve stiffness and cross-sectional area have been reported after a single session (Khademi et al. 2023). Nevertheless, these techniques are also used, albeit less frequently, in healthy or asymptomatic individuals (Neto et al. 2019). For example, a six-week program of neural gliding exercises was shown to be more effective than static stretching in improving hamstring flexibility (de Ridder et al. 2020). Furthermore, sliders appear to be more effective than tensioners in increasing hip flexion range of motion (Lim et al. 2021).

In line with these observations, the present study compared neurodynamic flossing and tensioning techniques with the commonly used static stretching. Our results first demonstrated a reduction in muscle stiffness, reflected by a decrease in biceps femoris SWV, regardless of the technique or stretching intensity. These findings are consistent with previous studies reporting reductions in muscle stiffness following static stretching. Several ultrasound-based studies have also shown an immediate decrease in muscle stiffness or shear modulus after stretching, reflecting an acute increase in muscle compliance (Nakamura et al. 2014).

The influence of stretching intensity on muscle mechanical properties remains controversial. While some studies suggested that higher intensities may induce greater reductions in muscle stiffness or larger increases in joint range of motion by inducing collagen fiber reorganization and enhanced viscoelastic stress relaxation (Kataura et al. 2017), others reported no additional benefit compared with moderate intensities (Bryant et al. 2023). Recent evidence suggested that stretching intensity is not a determining factor as long as the stretch is performed at a large joint angle (Warneke et al. 2025). Moreover, studies investigating stretches performed up to 120% of the tolerated range of motion indicated that excessive intensity may induce pain and compromise the desired mechanical adaptations, particularly reductions in muscle stiffness (Takeuchi et al. 2021). It may be due to protective neuromuscular responses associated with activation of nociceptive pathways (Takeuchi et al. 2021). The absence of significant differences in muscular parameters between static and neurodynamic techniques is therefore not surprising, as all conditions involved identical joint positions. However, a previous study using neurodynamic stretching did not report changes in muscle SWV (Andrade et al. 2018). These discrepancies may be explained by differences in stretching posture. Indeed, in our present study, an elongated position with exacerbated hip flexion and knee extension was used, whereas the previous studies used seated positions (Andrade et al. 2018).

In contrast, SWV measurements at the sciatic nerve level revealed distinct results. Indeed, only the maximal-intensity flossing condition (FLOS100) induced a decrease in sciatic nerve SWV. This finding is consistent with previous studies reporting neural tissue modifications assessed using shear wave elastography (Andrade et al. 2016; Ciuffreda et al. 2024; Cotteret et al. 2025). It confirmed the ability of neurodynamic mobilizations to promote neural gliding through surrounding connective tissues, thereby reducing peripheral mechanical constraints, particularly during large-amplitude flossing movements (Shacklock 1995; Coppieters and Butler 2008; Bueno-Gracia et al. 2020). This mechanism may also contribute to pain reduction by decreasing stress on the neural sheath. Our results also confirmed differences in neuromuscular responses between flossing and tensioning techniques, which are similar to previous evidences (Ellis et al. 2022). However, this selective neural tissue response is attributed to the distinct mechanical rationale of these two mobilization techniques. Indeed, tensioning relies on convergent joint movements, such as cervical flexion combined with ankle dorsiflexion, which apply a direct tensile load to the nerve and therefore promote neural elongation and substantial strain. Conversely, flossing-type mobilizations (sliders) are based on antagonistic or alternating movements (e.g. cervical extension with dorsiflexion, followed by cervical flexion with plantarflexion), which generate nerve excursion relative to the surrounding tissues without imposing a large tensile load on the neural structure itself. These alternating movements, often combined with muscle activation and joint displacement, may therefore promote neural gliding rather than stretching under sustained tension. The failure of maximal-intensity tensioning (TENS100) to reduce nerve stiffness despite applying sustained neural tension therefore suggested that elongation-based loading alone is insufficient for acute neural stiffness reductions in healthy tissue. Nerve mobilization appeared to be more important that a prolonged neural tension. This finding contrasts with the established effectiveness of tensioning in symptomatic populations where pathological stiffening and fibrosis limit neural accommodation. In asymptomatic tissue with intact mechanical compliance, sustained tension may not engage accommodation mechanisms; rather, it may prompt compensatory stiffening or resistance to load. This finding aligns with recent neurophysiological evidence (Alharmoodi et al. 2022) demonstrating that tensioning and gliding produce fundamentally different autonomic and central nervous system responses, suggesting distinct mechanotransduction pathways.

The superior effectiveness of maximal-intensity flossing was confirmed by the slump test results. Although improvements were observed across all stretching modalities, the largest gains were obtained in the FLOS100 condition. The slump test is widely used in both clinical and research settings to assess mechanosensitivity and neural flossing (gliding) capacity of the sciatic nerve (Majlesi et al. 2008). Improvements are generally interpreted as an increase in neural compliance. In the present study, the concomitant decrease in sciatic nerve SWV and increase in range of motion during the slump test support the notion that neurodynamic flossing performed at 100% of the maximal range of motion, in five one-minute sets at 30 bpm, promotes beneficial sciatic nerve excursion in healthy, physically active individuals. However, the superiority of neurodynamic stretching performed at a maximal range of motion remains questionable. Some authors have suggested that a moderate mechanical strain may promote neural tissue recovery by reducing intraneural fibrosis, facilitating remyelination, and modulating the immune response (Ellis et al. 2022). Conversely, excessive loading may be detrimental.

Despite the neural tissue changes observed in the FLOS100 condition, we did not observe superior improvements in flexibility compared with the other experimental conditions, as assessed using the PKE and SR tests. This finding may appear surprising since authors have suggested that neurodynamic techniques could induce greater effects than static stretching, particularly regarding hamstring flexibility (Satkunskiene et al. 2020). In contrast, numerous studies have reported increases in joint range of motion following different stretching modalities, regardless of the technique applied (Andrade et al. 2018; Behm et al. 2023; Konrad et al. 2024; De Arenas-Arroyo et al. 2025). Several methodological factors may explain the absence of between-condition differences. For example, authors have suggested that changes in spinal curvature, such as those occurring during forward bending, do not significantly influence sciatic nerve excursion (Ellis et al. 2017). Furthermore, the requirement for participants to change posture between the intervention and the flexibility assessments (from seated to standing) may have introduced variability and attenuated potential differences between conditions. Taken together, our results suggest that a decrease in sciatic nerve SWV does not necessarily translate into a greater immediate improvement in flexibility in healthy individuals compared with static stretching. This observation indicates that the sciatic nerve may contribute to the range of motion in specific limb positions, as reflected by the increased range of motion during the slump test, but not in global flexibility tests such as the SR and PKE.

In the present study, all stretching interventions have induced a significant decrease in maximal hamstring torque. This finding is consistent with previous literature. Indeed, most studies have shown that a prolonged static stretching, generally exceeding 60 s per set, results in a significant reduction in MVIC (Behm and Chaouachi 2011; Warneke et al. 2025). These impairments are commonly attributed to neural mechanisms, such as central inhibition or reduced motoneuron excitability (Trajano et al. 2014), as well as to muscular mechanisms, including alterations in the length-tension relationship (Warneke and Lohmann 2024). In our study, the neural contribution was illustrated by a significant decrease in EMG activity of the semitendinous muscle, whereas no change was observed in the biceps femoris. This dissociation may be attributed by architectural differences between these two muscles. Indeed, stretch sensitivity varies according to muscle fiber orientation and muscle-specific structural characteristics (Kellis and Blazevich 2022).

Our findings therefore support the hypothesis that prolonged stretching may reduce force production capacity. At first glance, this result may appear unexpected, as we anticipated that stretching interventions targeting neural tissue, particularly when performed at submaximal intensity, would not affect muscle force. However, the stretching positions used in the present protocol likely imposed greater mechanical strain on the muscle–tendon unit than on the sciatic nerve. It is possible that alternative joint configurations, particularly at the hip or ankle, are required to more specifically target neural tissue while limiting mechanical loading of the muscle–tendon complex. This could potentially be achieved through the inclusion of hip rotational components.

Moreover, substantial interindividual anatomical variability, particularly regarding the course, depth, and mechanical environment of the sciatic nerve, may have contributed to the heterogeneity of the observed responses. These differences, including the characteristics of surrounding tissues and the mechanical properties of the nerve itself, may partly explain the contradictory findings reported in the literature. In addition, individual responses to stretching may vary considerably depending on several factors, such as the type of technique applied, injury history, baseline flexibility (Babault et al. 2015), or specific mechanical constraints imposed on some structures, such as the tendon (Silvers-Granelli et al. 2021). These interindividual differences should be considered both in future experimental designs and in clinical applications.

### Limitations and perspectives

Several limitations should be acknowledged. First, only asymptomatic athletes were included in this study. Therefore, whether these findings can be generalized to symptomatic populations, particularly individuals presenting pain or musculoskeletal disorders, remains uncertain. Furthermore, the testing position used was not conventional and did not reflect a posture commonly adopted in field-based settings. Although SWE provides a precise and localized assessment of tissue stiffness, this technique reflects stiffness at a restricted anatomical site and does not allow for a global evaluation of the myotenidnous complex. Another limitation relates to the fact that neurodynamic flossing involves activation of muscles such as the tibialis anterior as well as cervical flexor and extensor muscles, which may induce muscle fatigue or a general warm-up effect, potentially acting as confounding factors. In addition, SWE measurements were performed only in a neutral joint position. Different results may have been observed if measurements had been conducted at maximal stretch positions, where both muscular and neural tissues are subjected to a greater mechanical strain. Finally, in light of the aforementioned limitations, it would be relevant to compare neurodynamic stretching techniques with other modalities, such as dynamic stretching, in order to determine whether neurodynamic mobilizations induce a warm-up effect comparable to that of more traditional stretching approaches.

## Conclusion

The present study indicates that all stretching modalities, irrespective of technique and intensity, resulted in an acute increase in flexibility along with a decrease in muscle stiffness. In contrast, only FLOS100 induced a reduction in sciatic nerve stiffness, suggesting a technique-specific effect on neural tissue. Additionally, all stretching interventions were associated with a reduction in hamstring force production capacity. This suggests the need to consider the potential neuromuscular consequences of stretching exercises. Taken together, these findings suggest that neurodynamic flossing may specifically target neural tissue. However, its immediate functional benefits appear dependent on joint position and biomechanical context. Further research is needed to determine the long-term effects and clinical relevance of neurodynamic mobilization techniques.

## Data Availability

Data are available on reasonable request to the corresponding author.

## Abbreviations

AKE: Active knee extension
BF: Muscle biceps femoris
CON: Control experimental condition
EMG: Electromyography
FLOS: Flossing neurodynamic stretching
MVIC: Maximal voluntary isometric contraction
PKE: Passive knee extension
ROM: Range of motion
SR: Stand and reach test
SS: Static stretching
ST: Muscle semitendinosus
SWE: Shear wave elastography
SWV: Shear wave velocity
TENS: Tensioning neurodynamic stretching
